# Measurement of ^131^I activity in thyroid of nuclear medical staff and internal dose assessment in a Polish nuclear medical hospital

**DOI:** 10.1007/s00411-016-0674-1

**Published:** 2016-12-31

**Authors:** K. Brudecki, A. Kowalska, P. Zagrodzki, A. Szczodry, T. Mroz, P. Janowski, J. W. Mietelski

**Affiliations:** 1 Institute of Nuclear Physics, Polish Academy of Sciences, Radzikowskiego 152, 31-342 Kraków, Poland; 2 Department of Endocrinology and Nuclear Medicine, Holy Cross Cancer Center, Artwińskiego 3, 25-734 Kielce, Poland; 3 Pedagogical University in Cracow, Podchorążych 2, 30-084 Kraków, Poland; 4 AGH University of Science and Technology, Mickiewicza 30, 30-059 Kraków, Poland; 5 Department of Food Chemistry and Nutrition, Medical College, Jagiellonian University, 30-688 Kraków, Poland

**Keywords:** ^131^I, Thyroid, Nuclear medicine, Medical personnel, Internal doses

## Abstract

This paper presents results of ^131^I thyroid activity measurements in 30 members of the nuclear medicine personnel of the Department of Endocrinology and Nuclear Medicine Holy Cross Cancer Centre in Kielce, Poland. A whole-body spectrometer equipped with two semiconductor gamma radiation detectors served as the basic research instrument. In ten out of 30 examined staff members, the determined ^131^I activity was found to be above the detection limit (DL = 5 Bq of ^131^I in the thyroid). The measured activities ranged from (5 ± 2) Bq to (217 ± 56) Bq. The highest activities in thyroids were detected for technical and cleaning personnel, whereas the lowest values were recorded for medical doctors. Having measured the activities, an attempt has been made to estimate the corresponding annual effective doses, which were found to range from 0.02 to 0.8 mSv. The highest annual equivalent doses have been found for thyroid, ranging from 0.4 to 15.4 mSv, detected for a cleaner and a technician, respectively. The maximum estimated effective dose corresponds to 32% of the annual background dose in Poland, and to circa 4% of the annual limit for the effective dose due to occupational exposure of 20 mSv per year, which is in compliance with the value recommended by the International Commission on Radiological Protection.

## Introduction

Iodine is one of the elements that are essential for proper functioning of the human organism. In adolescents, the regular iodine content varies between 30 and 50 mg (Venturi et al. 2000). The majority of iodine is stored in the thyroid gland, where an iodine–sodium symporter mechanism regulates iodine accumulation against the electrochemical gradient. The specific and natural iodine affinity of the thyroid proved extremely useful for nuclear medicine. While iodine has as many as 36 known unstable isotopes, ^131^I is the radioisotope most frequently applied in nuclear medicine. In fact, it was used for the first time in 1942 to treat hyperthyroidism (Seanger et al. 1979). Since then it has been widely applied both in diagnosis and treatment of thyroid-related illnesses. ^131^I is also used in kidney and bladder function tests.

At present, 42 staff members are employed at the Department of Endocrinology and Nuclear Medicine Holy Cross Cancer (E&NM HCC) in Kielce, Poland. The team consists of 25 nurses, nine medical doctors, two technicians and six cleaning personnel. ^131^I is applied by the staff both in thyroid scintigraphy and in hyperthyroidism and thyroid cancer treatment. In total, circa 1500 radio-iodine treatment sessions are performed annually. In general, medical staff dealing with nuclear medicine should be entirely safe in their professional environment. Nevertheless, their exposure to radioactive iodine seems to be partially uncontrolled while they perform their duties, namely while administering iodine in the form of tablets or solutions or while taking care of the patients already treated with the isotope. To verify such a hypothesis, ^131^I activity measurements in the thyroids of the personnel employed at the Department of E&NM HCC were performed.

## Material and method

### Medical staff

The present study included 30 employees of the Department of E&NM HCC in Kielce, Poland, which corresponds to circa 70% of all of the staff employed. ^131^I activities in two technicians, 19 nurses, three medical doctors and six cleaning personnel have been measured. The group in the study consisted of one man and 29 women. The man was 46 years old at the time of measurement, whereas the women were aged 36–58 years. The research was approved by the Bioethics Committee at the Regional Medical Chamber in Krakow.

### ^131^I activity measurements

^131^I thyroid activity measurements in the medical personnel were performed with the whole-body spectrometer at the Institute of Nuclear Physics, Polish Academy of Sciences in Krakow. The dimensions of the shielded spectrometer chamber are 2 m in length, 1.2 m in width and 1.3 m in height, and it is shielded both with a 17-cm-thick shield made of nineteenth-century steel and a 2-cm layer of electrolytic copper. The total mass of the shielding is 18 t. The spectrometer consists of two germanium detectors manufactured by Ortec, each characterised by a relative efficiency of 30% (Mietelski et al. 2013). Performance calibration was carried out with an adult thyroid phantom including a sodium iodide solution with an activity of ca 6 kBq (Kierepko et al. 2014). A phantom thyroid lobe with dimensions of 5.5 cm in length, 2.5 cm in width and 2 cm in height was applied. One hundred and fifty activity measurements were taken to determine the average performance of the detector at various phantom—detector geometries. Each measurement corresponded to a different phantom configuration within a 5-cm-wide, 5-cm-long and 3-cm-tall cuboid. The large number of measurements was necessary because of possible variations of thyroid location in a healthy human in relation to the detector position. The average detector efficiency was equal to (0.31 ± 0.08)%. The measurements were taken over 60 min, with the detector set perpendicularly to the patient thyroid at a distance of about 2 cm from the surface of the neck. With the chosen measurement time, a detection limit of ^131^I in the thyroid at the level of 5 Bq was obtained.

### Survey research

Each participant in the study was asked to complete a survey questionnaire. The questions can be divided into four categories. General questions such as gender and age formed the first ‘demographic’ category. The second category dealt with determining the nature of work with radioactive iodine including details on the type of tasks and ^131^I handling, duration of exposure to ^131^I and contacts with patients after treatment or diagnostics. The third category was concerned with dietary habits, whereas the fourth category addressed health conditions related to possible thyroid diseases. The responses provided by the medical personnel served to support interpretation of the ^131^I activity measured in the thyroid.

### Dose estimation

On the basis of the results obtained for ^131^I thyroid activity in the medical personnel, doses were estimated. The initial step was to determine the nature of the staff exposure. The exposure to ^131^I for Department of E&NM HCC employees shows a rather regular pattern. As for iodine, the effective half-life within a human body is approximately equal to 7 days (Kalinyak and McDougall 2003), which is relatively long in comparison with the occupational procedures that the employees perform. Thus, the thyroids of the subjects under examination should be ‘radioactively balanced’. Therefore, the iodine activities measured in the thyroids of employees were stable.

The next step to estimate the doses was to determine the professional routines for the medical staff under examination. Their routine schemes combined with the survey data have shown that the nurses in the therapeutic part of the department work one 12-h shift every 3 days. Therapeutic doses are administered to patients weekly on Fridays. In contrast, the cleaning staff cleans the rooms of the patients undergoing the treatment on a regular weekly basis, on Wednesdays.

**Fig. 1:**
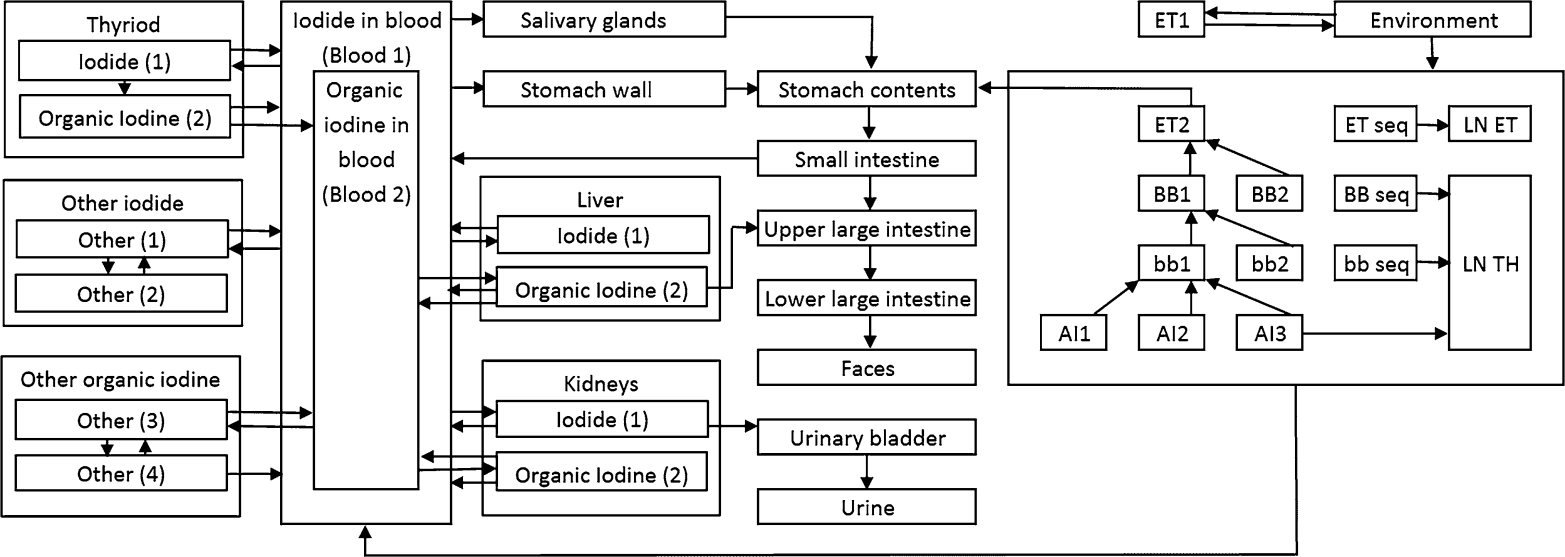
General biokinetic model applied in the present study. The model combines ICRP Human Respiratory Track Model, Gastro-Intestinal Tract Model and Leggett systemic model (ET1 extrathoracic region, ET2 posterior nasal passages, LN ET, TH lymph nodes, BB bronchial, bb bronchiolar, AI alveolar–interstitial)

Subsequently, for employees with ^131^I detected in their thyroids, the total iodine absorption in their respiratory system, and in other organs alike, was reconstructed. A weekly frequency of iodine intake was assumed for the cleaning and technical personnel, while for nurses a 3-day frequency was assumed. The iodine biokinetic model applied was that developed by Leggett (2010), combined with the Human Respiratory Tract Model (ICRP 1994; 2002) and Gastro-Intestinal Tract Model (ICRP 1979) developed by the International Commission on Radiological Protection (ICRP). The overall scheme for the model is shown in Fig. 1, whereas the biological transfer coefficients are presented in Table 1. SAAM II software by the Epsilon Group was applied for the biokinetic modelling (Barrett et al. 1998).

**Table 1:** Parameter values of the Leggett ^131^I systemic model (Leggett 2010)

Pathway	Transfer coefficient (day^−1^)
Blood 1 to thyroid 1	7.26
Blood 1 to urinary bladder contents	11.84
Blood 1 to salivary glands	5.16
Blood 1 to stomach wall	8.60
Blood 1 to other 1	600
Blood 1 to kidneys 1	25
Blood 1 to liver 1	15
Salivary glands to stomach contents	50
Stomach wall to stomach contents	50
Thyroid 1 to thyroid 2	95
Thyroid 1 to blood 1	36
Thyroid 2 to blood 2	0.0077
Thyroid 2 to blood 1	0
Other 1 to blood 1	330
Other 1 to other 2	35
Other 2 to other 1	56
Kidney 1 to blood 1	100
Liver 1 to blood 1	100
Blood 2 to other 3	15
Other 3 to blood 2	21
Other 3 to other 4	1.2
Other 4 to other 3	0.62
Other 4 to blood 1	0.14
Blood 2 to kidneys 2	3.6
Kidneys 2 to blood 2	21
Kidneys 2 to blood 1	0.14
Blood 2 to liver 2	21
Liver 2 to blood 2	21
Liver 2 to blood 1	0.14
Stomach contents to SI contents	20.57
SI contents to blood 1	594
Urinary bladder contents to urine	12

It was further assumed that the medical staff’s major exposure was due to iodine in the gaseous fraction (Mietelski et al. 2005). Elemental and organic iodine gas fractions are absorbed in the respiratory track in various proportions. However, whenever it is not possible to distinguish between elemental and organic iodine, it is recommended by ICRP to adopt a deposition rate for the elemental iodine. Therefore, for the present study 100% deposition in the respiratory tract was assumed, with the respective distribution of 10% in ET1 (anterior nose), 40% in ET2 (posterior nasal passage, larynx, pharynx and mouth) and 50% in BB (bronchial region) compartments (ICRP 1995).

Based on the measured ^131^I activity in the thyroid and the applied biokinetic models, effective annual doses as well as equivalent doses for particular organs were calculated: equivalent doses were calculated as based on the time-integrated ^131^I activity and radiation-weighted *S*_w_ factors. In practice, the time-integrated ^131^I activity was obtained from computer modelling and the radiation-weighted *S*_w_ factor was calculated with the SEECAL program (Oak Ridge National Laboratory, Oak Ridge, TN, USA), separately for adult men and women.

## Results and discussion

### ^131^I activity measurements

A total of 30 staff members in the Department of E&NM HCC in Kielce, Poland, were examined. ^131^I activity in thyroids was found to be below the detection limit (DL = 5 Bq of ^131^I in the thyroid) for 20 subjects in the study. Among the remaining 10 individuals, ^131^I thyroid activity ranged from (5 ± 2) to (217 ± 56) Bq for cleaning staff members and radiology technicians, respectively. For detailed results, see Table 2. The uncertainty of the presented results is about one sigma. ^131^I was detectable in the thyroid of all of the radiology technicians, half of the cleaning staff members and one-fourth of the nurses under examination, whereas it remained undetected for all medical doctors.

**Table 2:** Measured ^131^I thyroid activity

Subject code	Gender	Age	Profession	^131^I Thyroid activity [Bq]
T1	M	46	Technician	217 ± 56
T2	F	53	Technician	107 ± 28
C1	F	40	Cleaner	152 ± 40
C2	F	46	Cleaner	91 ± 24
C3	F	42	Cleaner	5 ± 2
C4	F	55	Cleaner	<5
C5	F	40	Cleaner	<5
C6	F	58	Cleaner	<4
N1	F	39	Nurse	66 ± 17
N2	F	44	Nurse	38 ± 10
N3	F	37	Nurse	38 ± 10
N4	F	46	Nurse	16 ± 5
N5	F	51	Nurse	15 ± 4
N6	F	40	Nurse	<5
N7	F	50	Nurse	<5
N8	F	46	Nurse	<6
N9	F	43	Nurse	<4
N10	F	41	Nurse	<5
N11	F	36	Nurse	<5
N12	F	50	Nurse	<5
N13	F	43	Nurse	<5
N14	F	49	Nurse	<5
N15	F	53	Nurse	<5
N16	F	38	Nurse	<5
N17	F	40	Nurse	<5
N18	F	45	Nurse	<5
N19	F	45	Nurse	<5
D1	F	44	Medical doctor	<5
D2	F	49	Medical doctor	<5
D3	F	52	Medical doctor	<5

This distribution pattern can be explained by the types of duties performed with respect to exposure to ^131^I: radiology technicians and radiopharmacists were found to be the most exposed contaminated individuals, because they directly participate in preparing and administering radiopharmaceuticals, where their presence at every treatment session is required. In contrast, the main duty of medical doctors is to collect medical surveys, order medical tests and provide advisory services, so their contact with ^131^I is limited. The least consistent results were obtained for the group of the cleaning staff and nurses. The Department of E&NM HCC in Kielce is divided into two parts, namely the diagnostic part and the therapeutic part. Relatively low activities are administered by the diagnostic division, and patients are not subjected to a strict isolation regime. This is different in the therapeutic division, where applied ^131^I activities tend to be relatively high and patients are completely isolated. It is noted that those nurses and cleaning staff members for whom ^131^I was detected had worked in the therapeutic division for over 1 month preceding the measurements.

### Modelling

The next stage of the study was to estimate doses for the medical staff members. The ^131^I total absorption into the respiratory system and ^131^I activity in the remaining organs were reconstructed by computer modelling, to match the measured ^131^I thyroid activity. The reconstructed results along with the parameters assumed for the dose calculations are presented in Table 3. A strong correlation was found between the simulated and measured thyroid activities (Pearson’s *R* ^2^ = 0.996, *p* = 0.000), although the simulated activities were significantly higher than the measured activities (Student’s *t* test for paired samples: *p* = 0.029).le 5

**Table 3:** Parameters assumed for dose computations

Subject code	Gender	Single absorption activity	Incorporation frequency	Number of incorporations over 1 year	Simulated ^131^I thyroid activity	Measured ^131^I thyroid activity
T1	M	750	Once a week	50	230	217 ± 56
T2	F	400	Once a week	50	120	107 ± 28
C1	F	500	Once a week	50	150	152 ± 40
C2	F	350	Once a week	50	100	91 ± 24
C3	F	15	Once a week	50	5	5 ± 2
N1	F	80	Every 3 days	112	70	66 ± 17
N2	F	50	Every 3 days	112	40	38 ± 10
N3	F	50	Every 3 days	112	40	38 ± 10
N4	F	20	Every 3 days	112	17	16 ± 5
N5	F	20	Every 3 days	112	17	15 ± 4

Based on the computer modelling data, the time-integrated ^131^I activity in the source organs was also calculated (Table 4).

**Table 4:** Time-integrated ^131^I activity in source organs

Medical staff/source organs	T1 (217 Bq)	T2 (107 Bq)	C1 (152 Bq	C2 (91 Bq)	C3 (5 Bq)	N1 (66 Bq)	N2, N3 (38 Bq)	N4, N5 (15 Bq, 16 Bq)
Thyroid	9.41 × 10^9^	5.02 × 10^9^	6.28 × 10^9^	4.39 × 10^9^	1.88 × 10^8^	2.24 × 10^9^	1.40 × 10^9^	5.61 × 10^8^
UBC	1.66 × 10^8^	8.84 × 10^7^	1.11 × 10^8^	7.74 × 10^7^	3.32 × 10^6^	3.92 × 10^7^	2.45 × 10^7^	9.80 × 10^6^
Kidneys	5.95 × 10^7^	3.17 × 10^7^	3.96 × 10^7^	2.77 × 10^7^	1.19 × 10^6^	1.41 × 10^7^	8.82 × 10^6^	3.53 × 10^6^
Blood	2.70 × 10^8^	1.44 × 10^8^	1.80 × 10^8^	1.26 × 10^8^	5.41 × 10^6^	6.42 × 10^7^	4.01 × 10^7^	1.61 × 10^7^
Liver	1.25 × 10^8^	6.66 × 10^7^	8.33 × 10^7^	5.83 × 10^7^	2.50 × 10^6^	2.99 × 10^7^	1.87 × 10^7^	7.47 × 10^6^
Other	6.71 × 10^8^	3.58 × 10^8^	4.47 × 10^8^	3.13 × 10^8^	1.34 × 10^7^	1.59 × 10^8^	9.94 × 10^7^	3.98 × 10^7^
Salivary glands	1.74 × 10^7^	9.30 × 10^6^	1.16 × 10^7^	8.14 × 10^6^	3.49 × 10^5^	4.12 × 10^6^	2.58 × 10^6^	1.03 × 10^6^
St contents	1.48 × 10^8^	7.90 × 10^7^	9.88 × 10^7^	6.91 × 10^7^	2.96 × 10^6^	3.50 × 10^7^	2.19 × 10^7^	8.76 × 10^6^
St wall	2.91 × 10^7^	1.55 × 10^7^	1.94 × 10^7^	1.36 × 10^7^	5.81 × 10^5^	6.87 × 10^6^	4.29 × 10^6^	1.72 × 10^6^
SI	5.08 × 10^6^	2.71 × 10^6^	3.39 × 10^6^	2.37 × 10^6^	1.02 × 10^5^	1.20 × 10^6^	7.50 × 10^5^	3.00 × 10^5^
ULI	2.04 × 10^7^	1.09 × 10^7^	1.36 × 10^7^	9.51 × 10^6^	4.08 × 10^5^	4.83 × 10^6^	3.02 × 10^6^	1.21 × 10^6^
LLI	3.38 × 10^7^	1.80 × 10^7^	2.25 × 10^7^	1.58 × 10^7^	6.75 × 10^5^	8.01 × 10^6^	5.00 × 10^6^	2.00 × 10^6^
ET1	3.04 × 10^8^	1.62 × 10^8^	2.03 × 10^8^	1.42 × 10^8^	6.09 × 10^6^	7.19 × 10^7^	4.50 × 10^7^	1.80 × 10^7^
ET2	7.36 × 10^6^	3.92 × 10^6^	4.90 × 10^6^	3.43 × 10^6^	1.47 × 10^5^	1.74 × 10^6^	1.09 × 10^6^	4.35 × 10^5^
BB	1.50 × 10^7^	8.01 × 10^6^	1.00 × 10^7^	7.00 × 10^6^	3.00 × 10^5^	3.55 × 10^6^	2.22 × 10^6^	8.87 × 10^5^

*UBC* urinary bladder contents, *St* stomach, *SI* small intestine, *ULI* upper large intestine, *LLI* lower large intestine, *ET1* anterior nose, *ET2* posterior nasal passage, larynx, pharynx and mouth, *BB* the bronchial region

### Dose estimation

The estimated effective annual dose received by the medical staff in the study was found to vary between 0.02 and 0.8 mSv per year. Not surprisingly, the highest annual equivalent organ doses were found for the thyroid, ranging from 0.4 to 15.4 mSv, as obtained for a cleaner and a technician, respectively (Table 5).

**Table 5:** Calculated annual organ equivalent and effective doses (given in Sv)

Medical staff/organs	T1 (217 Bq)	T2 (107 Bq)	C1 (152 Bq)	C2 (91 Bq)	C3 (5 Bq)	N1 (66 Bq)	N2, N3 (38 Bq)	N4, N5 (15 Bq, 16 Bq)
Adrenals	1.5 × 10^−6^	1.0 × 10^−6^	1.3 × 10^−6^	8.9 × 10^−7^	3.8 × 10^−8^	4.5 × 10^−7^	2.8 × 10^−7^	1.1 × 10^−7^
Bladder wall	2.6 × 10^−5^	1.8 × 10^−5^	2.3 × 10^−5^	1.6 × 10^−5^	6.8 × 10^−7^	8.0 × 10^−6^	5.0 × 10^−6^	2.0 × 10^−6^
Bone surfaces	4.3 × 10^−6^	2.6 × 10^−6^	3.2 × 10^−6^	2.2 × 10^−6^	9.6 × 10^−8^	1.1 × 10^−6^	7.1 × 10^−7^	2.8 × 10^−7^
Brain	4.8 × 10^−6^	2.7 × 10^−6^	3.4 × 10^−6^	2.4 × 10^−6^	1.0 × 10^−7^	1.2 × 10^−6^	7.5 × 10^−7^	3.0 × 10^−7^
Breasts	1.7 × 10^−6^	1.2 × 10^−6^	1.5 × 10^−6^	1.0 × 10^−6^	4.4 × 10^−8^	5.2 × 10^−7^	3.2 × 10^−7^	1.3 × 10^−7^
St wall	1.2 × 10^−5^	6.9 × 10^−6^	8.6 × 10^−6^	6.0 × 10^−6^	2.6 × 10^−7^	3.1 × 10^−6^	1.9 × 10^−6^	7.6 × 10^−7^
SI Wall	1.2 × 10^−6^	8.1 × 10^−7^	1.0 × 10^−6^	7.1 × 10^−7^	3.0 × 10^−8^	3.6 × 10^−7^	2.3 × 10^−7^	9.0 × 10^−8^
ULI wall	2.7 × 10^−6^	1.7 × 10^−6^	2.1 × 10^−6^	1.5 × 10^−6^	6.2 × 10^−8^	7.4 × 10^−7^	4.6 × 10^−7^	1.8 × 10^−7^
LLI wall	5.4 × 10^−6^	3.0 × 10^−6^	3.8 × 10^−6^	2.6 × 10^−6^	1.1 × 10^−7^	1.3 × 10^−6^	8.4 × 10^−7^	3.3 × 10^−7^
Kidneys	7.6 × 10^−6^	4.6 × 10^−6^	5.8 × 10^−6^	4.0 × 10^−6^	1.7 × 10^−7^	2.1 × 10^−6^	1.3 × 10^−6^	5.1 × 10^−7^
Liver	3.7 × 10^−6^	2.5 × 10^−6^	3.1 × 10^−6^	2.2 × 10^−6^	9.4 × 10^−8^	1.1 × 10^−6^	7.0 × 10^−7^	2.8 × 10^−7^
Lungs	2.5 × 10^−5^	1.5 × 10^−5^	1.9 × 10^−5^	1.3 × 10^−5^	5.6 × 10^−7^	6.6 × 10^−6^	4.1 × 10^−6^	1.7 × 10^−6^
ET airways	6.9 × 10^−5^	4.7 × 10^−5^	5.3 × 10^−5^	3.7 × 10^−5^	1.6 × 10^−6^	1.9 × 10^−5^	1.2 × 10^−5^	4.7 × 10^−6^
Muscle	4.2 × 10^−6^	2.9 × 10^−6^	3.6 × 10^−6^	2.5 × 10^−6^	1.1 × 10^−7^	1.3 × 10^−6^	8.0 × 10^−7^	3.2 × 10^−7^
Ovaries	^–^	8.6 × 10^−7^	1.1 × 10^−6^	7.6 × 10^−7^	3.2 × 10^−8^	3.8 × 10^−7^	2.4 × 10^−7^	9.6 × 10^−8^
Pancreas	1.9 × 10^−6^	1.2 × 10^−6^	1.5 × 10^−6^	1.0 × 10^−6^	4.5 × 10^−8^	5.3 × 10^−7^	3.3 × 10^−7^	1.3 × 10^−7^
Red marrow	3.2 × 10^−6^	2.0 × 10^−6^	2.5 × 10^−6^	1.7 × 10^−6^	7.4 × 10^−8^	8.9 × 10^−7^	5.5 × 10^−7^	2.2 × 10^−7^
Skin	2.1 × 10^−6^	1.3 × 10^−6^	1.7 × 10^−6^	1.2 × 10^−6^	5.0 × 10^−8^	6.0 × 10^−7^	3.7 × 10^−7^	1.5 × 10^−7^
Spleen	1.5 × 10^−6^	1.0 × 10^−6^	1.3 × 10^−6^	9.2 × 10^−7^	3.9 × 10^−8^	4.7 × 10^−7^	2.9 × 10^−7^	1.2 × 10^−7^
Testes	9.3 × 10^−7^	–	–	–	–	–	–	–
Thymus	5.1 × 10^−6^	4.1 × 10^−6^	5.2 × 10^−6^	3.6 × 10^−6^	1.6 × 10^−7^	1.8 × 10^−6^	1.2 × 10^−6^	4.6 × 10^−7^
Thyroid	1.5 × 10^−2^	9.7 × 10^−3^	1.2 × 10^−2^	8.5 × 10^−3^	3.6 × 10^−4^	4.3 × 10^−3^	2.7 × 10^−3^	1.1 × 10^−3^
GB wall	1.5 × 10^−6^	9.6 × 10^−7^	1.2 × 10^−6^	8.4 × 10^−7^	3.6 × 10^−8^	4.2 × 10^−7^	2.7 × 10^−7^	1.1 × 10^−7^
HT wall	2.3 × 10^−6^	1.5 × 10^−6^	1.9 × 10^−6^	1.3 × 10^−6^	5.6 × 10^−8^	6.6 × 10^−7^	4.1 × 10^−7^	1.7 × 10^−7^
Uterus	1.5 × 10^−6^	9.8 × 10^−7^	1.2 × 10^−6^	8.6 × 10^−7^	3.7 × 10^−8^	4.3 × 10^−7^	2.7 × 10^−7^	1.1 × 10^−7^
Remainder	4.2 × 10^−6^	2.8 × 10^−6^	3.5 × 10^−6^	2.5 × 10^−6^	1.1 × 10^−7^	1.3 × 10^−6^	7.8 × 10^−7^	3.1 × 10^−7^
Colon	3.9 × 10^−6^	2.2 × 10^−6^	2.8 × 10^−6^	2.0 × 10^−6^	8.4 × 10^−8^	1.0 × 10^−6^	6.2 × 10^−7^	2.5 × 10^−7^
Effective dose	7.8 × 10^−4^	4.9 × 10^−4^	6.1 × 10^−4^	4.3 × 10^−4^	1.8 × 10^−5^	2.2 × 10^−4^	1.4 × 10^−4^	5.4 × 10^−5^

*St* stomach, *SI* small intestine, *ULI* upper large intestine, *LLI* lower large intestine, *ET* extrathoracic, *GB* gallbladder, *HT* heart

While using radiopharmaceuticals for therapeutic or diagnostic purposes in medicine, appropriate radiological safety rules should be observed and proper safety measures taken. Exposure risk assessment methodologies to be applied to medical staff usually take into account only the external exposure to radiation, and as such are based either on readings from individual thermoluminescent dosimeters or on measurements performed either with scintillation probes or Geiger counters (Chiesa et al. 1997; Lancelot et al. 2008). Doses estimated in that way determine the exposure of medical staff dealing with ^131^I, ^99m^Tc, ^18^F, ^90^Y and ^153^Sm isotopes. Effective doses for medical staff determined for most of the singular medical procedures varied within the range (0.2–0.4) μSv per treatment. While administering treatment units of ^131^I-lipiodol, the annual dose for hands and chest of medical staff varied within the range from 140 to 443 μSv and from 12 to 23 μSv (Garin et al. 2003), respectively, whereas in another study the annual doses for hands and whole body were 165 and 3.8 μSv (Lancelot et al. 2008), respectively.

The methodology applied in the present study of measuring internal exposure is rarely applied. Similar studies have also been carried out at three hospitals in Warsaw (Poland). The reported levels of activities and doses were similar to those presented here, typically falling for effective dose within the range from 0.21 to 1.44 mSv per year (Krajewska and Pachocki 2013).

It is emphasised that such doses do not exceed the doses set out in the 2013/59 Euroatom (2014) directive, where the limit of the effective dose for occupational exposure is 20 mSv per year. However, under special circumstances, or for certain exposure situations specified in the national legislation, a higher effective dose, specifically up to 50 mSv, may be authorised by a competent authority for a certain year, provided that the average annual dose over five consecutive years, including the year(s) for which the limit has been exceeded, does not exceed 20 mSv. The same limits for occupational exposure are recommended by ICRP (2007) and required by Polish law.

## Conclusions

The maximum effective dose estimated in the present study for medical staff handling with ^131^I corresponds to 32% of the mean annual background effective dose due to natural sources in Poland, i.e. 2.48 mSv per year (Janik and Tokonami 2009). Moreover, the maximum estimated effective dose found here for medical staff was significantly below the annual limit set for occupational exposure, compliant with the value of 20 mSv recommended by ICRP (2007). Consequently, any negative health impact due to such doses is unlikely for the medical staff under examination. Nevertheless, the dose equivalent values estimated in the present study for the thyroid provide a worrying picture, because the dose equivalents determined for the cleaning staff members and the radiopharmaceutical technician were equal to 0.4 and 15.4 mSv, respectively.

Currently, at Polish nuclear medicine units only the staff exposure to external radiation is monitored. Measurements by thermoluminescent dosimeters, however, will not provide any information on the doses due to radio-iodine incorporated into the body. Therefore, the radiological safety standards applied at present fail to meet the requirements of the so-called conservative assessment rule, where upper dose limits should be estimated. With internal dose estimation for the incorporated radio-iodine entirely ignored, the assessed doses tend to be underestimated, and the doses due to the radionuclides absorbed into the body remain undetermined. Therefore, periodic and systematic monitoring of the internal contamination should be integrated into radiological protection standards for teams dealing with highly radioactive ^131^I.
